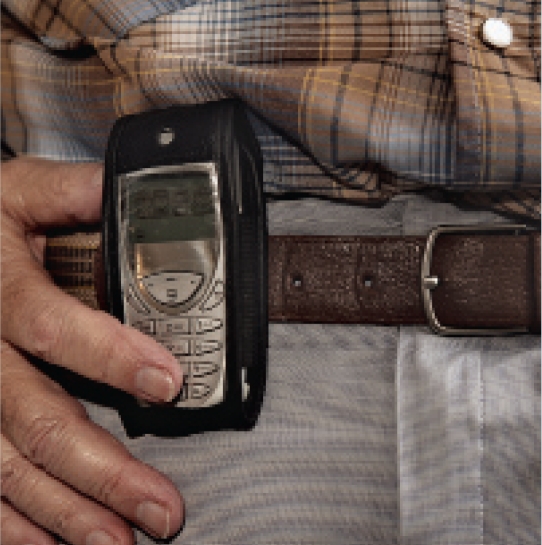# The Beat

**Published:** 2009-12

**Authors:** Erin E. Dooley

## Young and Restless

In work published online 3 Nov 2009 in the *Journal of Epidemiology and Community Health*, sons of women who reported smoking heavily in pregnancy were nearly twice as likely as sons of nonsmokers to exhibit restlessness and distractibility at age 3 years. Despite limitations—potential underreporting of smoking in pregnancy, dependence on parental reports of behavior, and unmeasured maternal characteristics that could influence child behavior—the study supports earlier animal findings that cigarette smoke exposure *in utero* affected neurologic development in fetuses. Jayne Hutchinson and colleagues write that studies in this cohort using teacher assessments of behavior at older ages may yield valuable insights.

**Figure:**
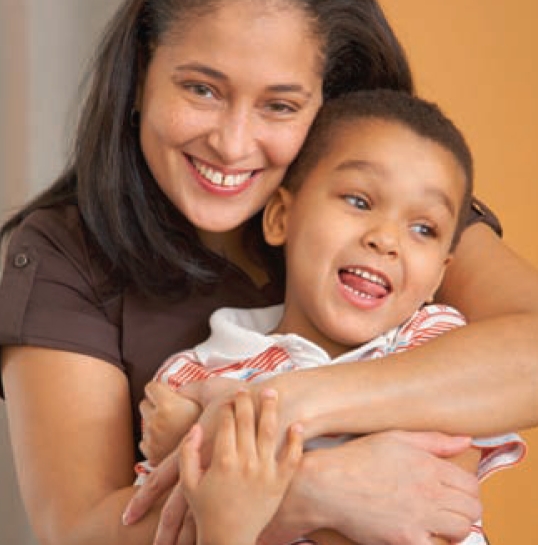


## Nanotech Summit Highlights Business Needs

Research Triangle Park, North Carolina, played host to the Research Triangle Environmental Health Collaborative’s second annual summit in October 2009. The summit focused on environmentally responsible development of nanotechnology, including critical environmental health issues faced by businesses in the development and manufacturing of nanomaterials. A guidance document with recommendations for business and policy makers will be forthcoming.

**Figure:**
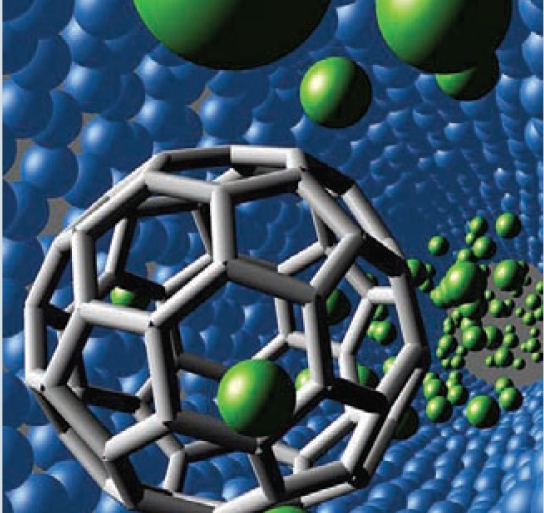


## Acetaminophen May Spur Asthma

A review in the Nov 2009 issue of *Chest* indicates acetaminophen use may be associated with as much as a 75% increased risk of developing asthma and wheeze in adults and a 60% increase in children. Senior author J. Mark FitzGerald and colleagues are attempting to help explain why asthma rates have risen over the past 30 years. Over this same period physicians began advising patients to use acetaminophen rather than aspirin because of aspirin’s link to Reye syndrome. This life‐threatening disorder can affect people of all ages, although it is perhaps most notorious for affecting children. Further prospective studies are required to better understand the acetaminophen–asthma connection, and the authors do not recommend abandoning acetaminophen as a treatment for flu symptoms in children.

## Sensor for Pesticides in Foods

In the 1 Nov 2009 issue of *Analytical Chemistry*, Zakir Hossain and colleagues describe a new biosensor they have developed that works more quickly and cheaply than conventional methods to detect small amounts of organophosphate and carbamate pesticides in foods and beverages. Conventional methods can take hours to reveal such contaminants, but the new bioactive paper sensor provides results in minutes. The researchers note their method could be especially useful in developing countries, which often lack access to electricity and expensive testing equipment.

## The Temperature of Conversion

A new study reveals that most land use changes in the United States lead to local and regional increases in surface temperature, with the greatest increases occurring with urbanization and conversion to bare soil. But Souleymane Fall and colleagues also report that conversion of land to agricultural uses resulted in cooler temperatures even if the land was previously forested, perhaps because of increased evaporation. These findings add to a growing body of knowledge that highlights the necessity of incorporating land use changes into climate change models. The paper appeared online 24 Aug 2009 ahead of print in the *International Journal of Climatology*.

## Phones and Bones

In a study of 150 male cell phone users, Tolga Atay and colleagues found that wearing a belt‐mounted phone was associated with decreased bone density in the pelvic iliac wing closest to the phone—perhaps, they suggest, due to exposure to electromagnetic fields (EMFs). Although the reduction in bone density was not statistically significant, the authors note the men in their study were relatively young (21–57 years old). If the reductions resulted from exposure to EMFs from the phones, the effect could grow with continued use. (Conversely, very weak EMFs have been used successfully to stimulate healing in broken bones.) The report appeared in the Sep 2009 issue of *The Journal of Craniofacial Surgery*.

**Figure:**